# Manufacturing and Testing of Radio Frequency MEMS Switches Using the Complementary Metal Oxide Semiconductor Process

**DOI:** 10.3390/s21041396

**Published:** 2021-02-17

**Authors:** Zung-You Tsai, Po-Jen Shih, Yao-Chuan Tsai, Ching-Liang Dai

**Affiliations:** 1 Department of Mechanical Engineering, National Chung Hsing University, Taichung 402, Taiwan; g9761407@mail.nchu.edu.tw; 2 Department of Biomedical Engineering, National Taiwan University, Taipei 106, Taiwan; pjshih@ntu.edu.tw; 3 Department of Bio-Industrial Mechatronics Engineering, National Chung Hsing University, Taichung 402, Taiwan; yctsaii@dragon.nchu.edu.tw

**Keywords:** radio frequency switch, capacitive shunt type, CMOS process, CMOS-MEMS technology

## Abstract

A radio frequency microelectromechanical system switch (MSS) manufactured by the complementary metal oxide semiconductor (CMOS) process is presented. The MSS is a capacitive shunt type. Structure for the MSS consists of coplanar waveguide (CPW) lines, a membrane, and springs. The membrane locates over the CPW lines. The surface of signal line for the CPW has a silicon dioxide dielectric layer. The fabrication of the MSS contains a CMOS process and a post-process. The MSS has a sacrificial oxide layer after the CMOS process. In the post-processing, a wet etching of buffer oxide etch (BOE) etchant is employed to etch the sacrificial oxide layer, so that the membrane is released. Actuation voltage for the MSS is simulated using the CoventorWare software. The springs have a low stiffness, so that the actuation voltage reduces. The measured results reveal that actuation voltage for the MSS is 10 V. Insertion loss for the MSS is 0.9 dB at 41 GHz and isolation for the MSS is 30 dB at 41 GHz.

## 1. Introduction

Radio frequency microelectromechanical system switches (MSSs) are higher performances in high frequency than solid-state switches [1]. The MSSs switches have been applied in reconfigurable antenna [1], microelectromechanical system (MEMS) satellites [2], wireless sensors [3], switchable bandpass filters [4], power amplifier [5], and receiver endoluminal coils [6]. Several radio frequency microelectromechanical system switches were developed utilizing silicon micromachining technology. For instance, Benoit et al. [7] used surface micromachining to develop a MSS on silicon-on-sapphire substrate. The MSS was actuated using a piezoelectric actuator, and the material of actuator was lead zirconium titanate. The insertion loss (IL) for the MSS was 1.4 dB at 67 GHz. Chae et al. [8] fabricated a hybrid MSS using surface micromachining. The hybrid MSS contained two actuators to reduce actuation voltage (AV). One actuator was driven by electrothermal force, and the other one was driven by electrostatic force. The electrothermal actuator needed an AV of 0.3 V. The AV of electrostatic actuator was 15.4 V. The hybrid MSS isolation was 38.8 dB at 2.4 GHz. Swarnkar et al. [9] employed surface micromachining to fabricate a MSS with two beams over the signal line of coplanar waveguide (CPW). The structure of two beams could reduce resistance and enhance isolation for the MSS at resonant frequency. The material of CPW was Cr/Au, and two beams were made by electroplating gold. The AV for the MSS was 20 V. The MSS isolation was 30 dB at 30 GHz, and the IL for the MSS was 0.5 dB at 30 GHz. Mafinejad et al. [10] proposed a radio frequency MSS with a flat and smooth beam. The erosion and bulge of beam surface influenced the performance of isolation and AV for MSS. The flat and smooth beam for the MSS was manufactured, so that AV reduced, and isolation increased. The MSS had an isolation of 20 dB at 21 GHz. The AV for the MSS was 18 V. Anuroop et al. [11] presented a MSS manufactured using surface micromachining and a packaging technique. A silicon cavity was fabricated using bulk micromachining. The MSS was bonded with the silicon cavity using an epoxy bonding. The packaging could protect the MSS to avoid environmental interference and improve electrical parasitic. The measurement showed that the MSS isolation was 19.57 dB at 10.9 GHz. Savin et al. [12] developed a radio frequency MSS. A packaging technique was used to bond the MSS and a quartz cap. The packaged MSS could isolate the outer environmental influence. The AV of the packaged MSS was 42 V. The MSS isolation was 30 dB at 20 GHz. Naito et al. [13] utilized surface micromachining to make a MSS with an actuator, and the actuator was composed of triple movable electrodes. The triple movable electrodes that actuated by electrostatic force controlled the MSS in "on" state or "off" state. The experiment showed that structure of the triple electrodes reduced AV and switching time for the MSS. The AV for the MSS was 9 V and it switching time was 5 μs. Rao et al. [14] manufactured a capacitive shunt MSS using surface micromachining. The membrane for the MSS was gold metal, and a dielectric layer of AlN located under the membrane. The AlN layer had a high dielectric constant, so that the MSS isolation increased. The AV for the MSS was 5.5 V. The MSS had an isolation of 72.4 dB at 27 GHz. Liu et al. [15] proposed a radio frequency MSS with a shunt protection metal. The shunt protection metal was used to enhance the MSS lifetime and increase the MSS isolation. The AV for the MSS was 60 V. The MSS isolation was 36 dB at 40 GHz. Lin et al. [16] employed the complementary metal oxide semiconductor (CMOS) process to develop a MSS with four inductors. The inductors were used to enhance the electrical properties for the MSS. The AV for the MSS was 11 V. The MSS isolation was 19 dB at 36 GHz. Yang et al. [17] also developed a MSS with inductors using the CMOS process. The inductors increased the isolation for the MSS. The MSS isolation was 25 dB at 35 GHz. The AV for the MSS was 11 V.

The CMOS-MEMS technology was used to develop microactuators [18,19,20,21], microsensors [22,23,24,25], and microgenerators [26]. Micro devices developed by this technology have a potential to mass-production by silicon manufacturing foundry. A MSS in this study was made using the CMOS-MEMS technology. The MSS fabricated by this technology is simpler than these MSSs [7,8,9,10,11,12,13,14,15] fabricated by surface micromachining. The isolation of the MSS is higher than that of Lin et al. [16] and Yang et al. [17], and the AV of the MSS is lower than that of Lin et al. [16] and Yang et al. [17].

## 2. Structure of MSS

**Figure 1:**
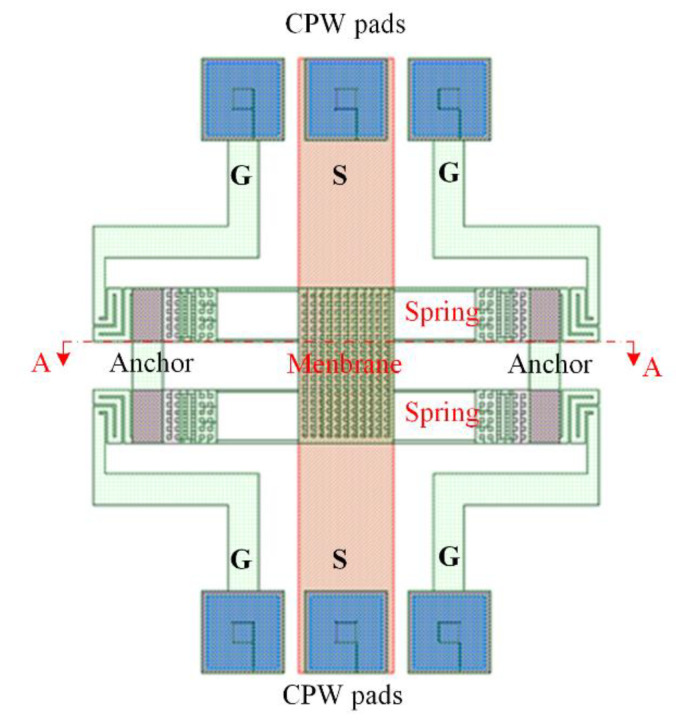
Microelectromechanical system switch (MSS) structure.

Figure 1 shows the MSS structure, where CPW is coplanar waveguide; G is ground line of CPW, and S is signal line of CPW. The MSS contains a membrane, CPW lines, springs, and anchors. The membrane is located over the CPW lines. Eight springs connect the membrane and anchors. Materials of the membrane and springs are the aluminum metal in the CMOS process. Materials of the coplanar waveguide lines are also the aluminum metal in the CMOS process. Width and thickness of the signal (S) line for CPW are 90 μm and 0.6 μm, respectively. Width and thickness of the ground lines for CPW are 30 μm and 0.6, respectively. The area of membrane is 92 × 150 μm^2^. Length, width, and thickness of each spring are 100 μm, 4 μm and 1 μm, respectively. 

**Figure 2:**
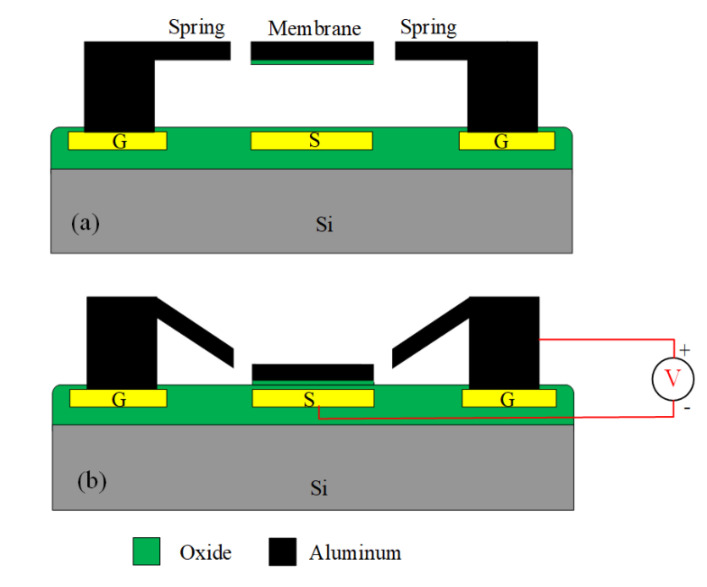
Cross-sectional view for the MSS: (**a**) in the on state; (**b**) in the off state.

The MSS is driven by an electrostatic force. Figure 2 shows the cross-sectional view of the MSS along the AA line (in Figure 1). As shown in Figure 2a, when the MSS is without AV, the membrane for the MSS keeps in the upper position. The MSS is in the on state, so that a radio frequency signal can propagate in the S line of CPW. As shown in Figure 2b, when an AV applies to the MSS, an electrostatic force is generated to actuate the membrane for the MSS, leading to the membrane moves down to the S line of CPW. The MSS is in the off state. The radio frequency propagation in the S line of CPW is coupled to the ground lines. The actuation voltage ($V_{av}$) of the MSS is given by Rao et al. [14],

(1) $$V_{av}=\sqrt{\frac{8k}{27\varepsilon_{0}Ag_{0}^{3}}}$$

where *k* is the total stiffness of springs, $\varepsilon_{0}$ is the dielectric constant of air, *A* is the overlap area of the membrane over the S line of CPW, and $g_{0}$ is the gap between the membrane and the S line. Each spring is a cantilever beam. The MSS has eight springs. The total stiffness of springs for the MSS is given by Rao et al. [14],

(2) $$k=\frac{24EI}{l^{3}}$$

where *E* is the Young’s modulus of beam, *I* is the moment of inertia for the beam and *l* is the length of beam. 

The radio frequency capacitive MSS provides high isolation at resonant frequency in the off state. The resonant frequency ($f_{0}$) can be expressed as [9],

(3) $$f_{0}=\frac{1}{2\pi \sqrt{L_{s}C_{s}}}$$

where $L_{s}$ is inductance of the membrane and springs and $C_{s}$ is capacitance between the membrane and the S line in the off state. The capacitance ($L_{s}$) relies on the geometry of the membrane and springs. The inductance ($C_{s}$) is determined by the overlap area of the membrane over the S line of CPW.

**Figure 3:**
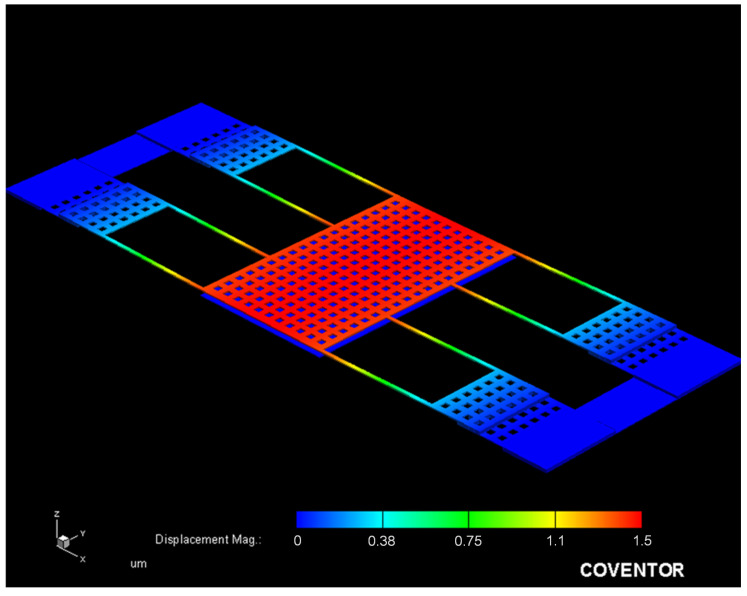
Simulation of membrane displacement for the MSS.

**Figure 4:**
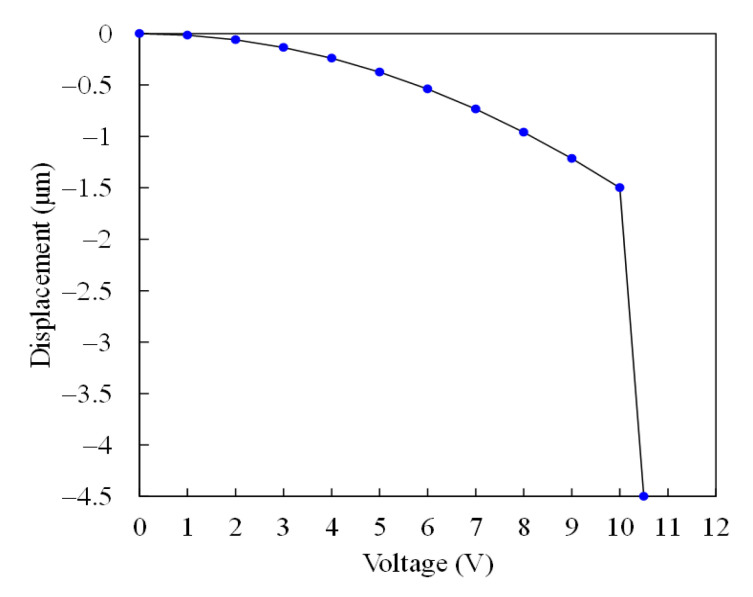
Membrane displacement vs. actuation voltage.

To character actuation voltage for the MSS, a finite element method (FEM) software CoventorWare is used to evaluate relation between of the membrane displacement and the applying voltage. According to the MSS structure as shown in Figure 1, the MSS model is constructed. The triangular element is used to mesh the MSS model. Materials of membrane and springs for the MSS are the aluminum metal in the CMOS process. Young’s modulus for aluminum is 70 GPa, and Poisson’s ratio for aluminum is 0.3. Mass density for aluminum is 2679 kg/m^3^ [27,28]. The above material parameters input the CoventorWare simulation system. An applying voltage of 10 V is applied to the MSS. Figure 3 shows the simulation of membrane displacement for the MSS. The simulation shows that the membrane has a displacement of 1.5 μm under an applying voltage of 10 V. Distance between the membrane and the S line for the MSS is 4.5 μm. To understand the AV for the MSS, the different voltages are applied to the MSS. Figure 4 shows the displacement of membrane under different voltages. In the investigation, the applying voltage varies from zero to 10.5 V. The results show that the membrane displacement moves down 4.5 μm at 10.5 V. Thereby, the AV for the MSS is 10.5 V.

**Figure 5:**
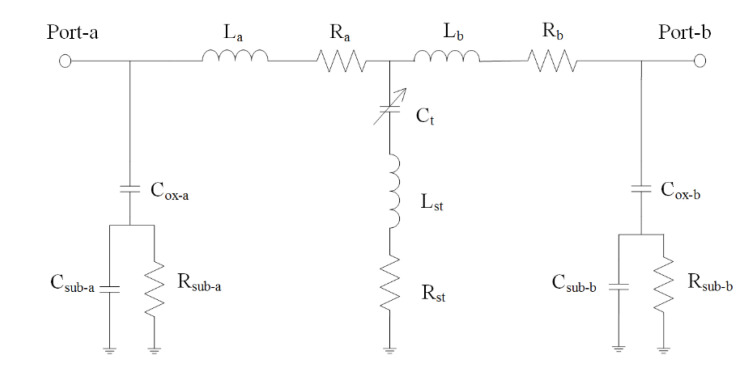
Equivalent circuit.

**Figure 6:**
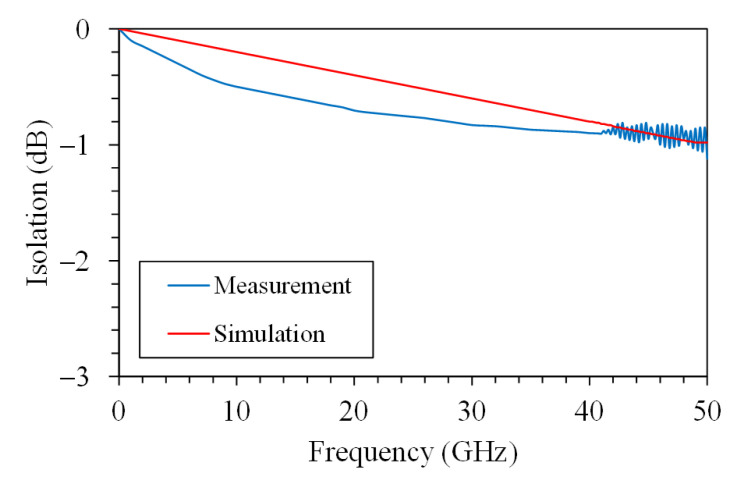
Insertion loss for the MSS.

**Figure 7:**
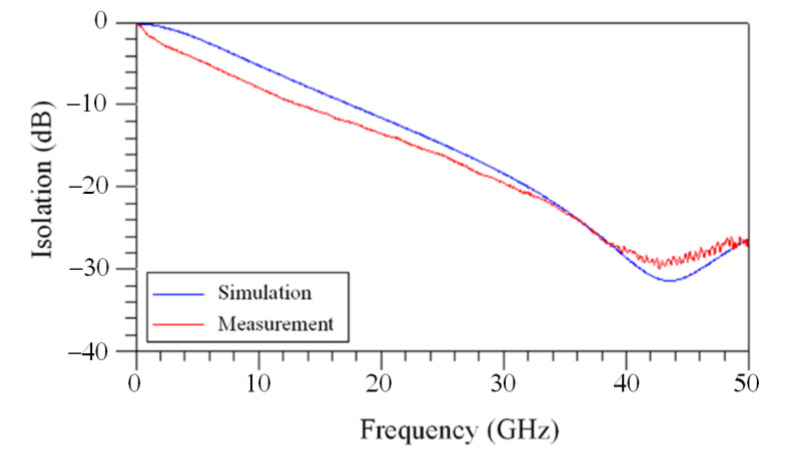
Isolation for the MSS.

Figure 5 presents equivalent circuit for the MSS [17], where port-a and port-b are input and output port for the MSS, respectively; L_a_ and L_b_ are inductor of the S line; R_a_ and R_b_ are resistance of the S line; C_sub-a_ and C_sub-b_ are capacitance of silicon substrate; R_sub-a_ and R_sub-b_ are resistance of silicon substrate; C_ox-a_ and C_ox-b_ are the insulated capacitance under the S line; C_t_ is capacitance between the membrane and the S line; R_st_ is resistance of the springs and the membrane; L_st_ is inductance of the springs and the membrane. The isolation and IL are important property for the MSS. An Ansoft Q3D extractor and an Agilent ADS tool are employed to evaluate the isolation and IL for the MSS [21]. According to the dimensions for the MSS, the Ansoft Q3D extractor is used to evaluate the electrical parameter for the MSS. The electrical parameters contain the inductor and resistance of the S line, the capacitance and resistance of the silicon substrate, the capacitance between the membrane and the S line, and the resistance and inductance of the springs and the membrane. The evaluated results show that the electrical parameters for the MSS are R_a_ = 0.042 Ω, R_b_ = 0.042 Ω, L_a_ = 29 pH, L_b_ = 29 pH, C_t_ = 0.87 pF, L_st_ = 36 pH, R_st_ = 0.51 Ω, R_sub-a_ = 250 Ω, C_sub-a_ = 28 fF, C_ox-a_ = 36 fF, R_sub-b_ = 250 Ω, C_sub-b_ = 28 fF and C_ox-b_ = 36 fF. The impedance of the CPW lines is 50 Ω. The above electrical parameters for the MSS are entered into the Agilent ADS to calculus the IL and isolation. Figure 6 shows the simulated IL for the MSS. The results show that the simulated IL for the MSS is 0.8 dB at 41 dB. Figure 7 shows the simulated isolation for the MSS. The simulated results show that the MSS have an isolation of 32 dB at 41 GHz.

## 3. Fabrication of MMS

**Figure 8:**
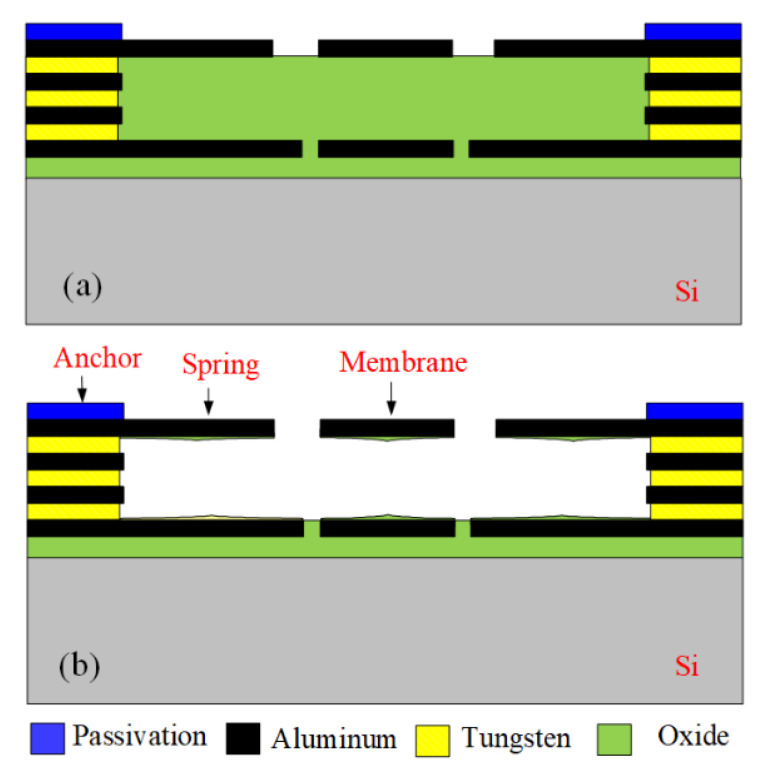
Process flow of the MSS: (**a**) after the complementary metal oxide semiconductor (CMOS) process; and (**b**) etching oxide layer.

**Figure 9:**
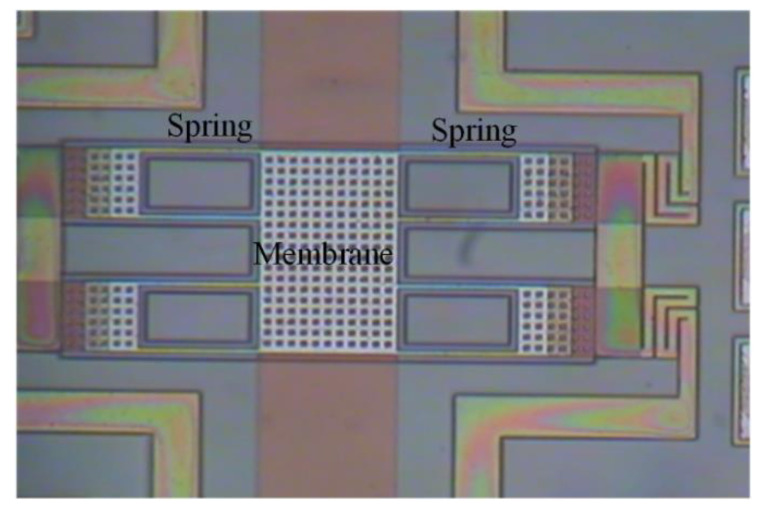
Optical picture of the MSS after the CMOS process.

The CMOS process and a post-process were used to make the MSS. Figure 8 illustrates the MSS process flow. First, the Taiwan Semiconductor Manufacturing Company (TSMC) in accordance with the structure layout for the MSS as shown in Figure 1 employs a commercial CMOS process to manufacture the MSS. An optical microscope (Microtech LX500-M, M&T Optic Co. Ltd., Taiwan) was used to take the MSS image. Figure 9 shows an optical image for the MSS after the CMOS process. At this stage, the membrane and springs as shown in Figure 9 are not suspension structures because they are fixed by a sacrificial oxide layer. In order to obtain the suspension structures of the membrane and spring, the MSS requires a post-processing to remove the sacrificial oxide layer.

**Figure 10:**
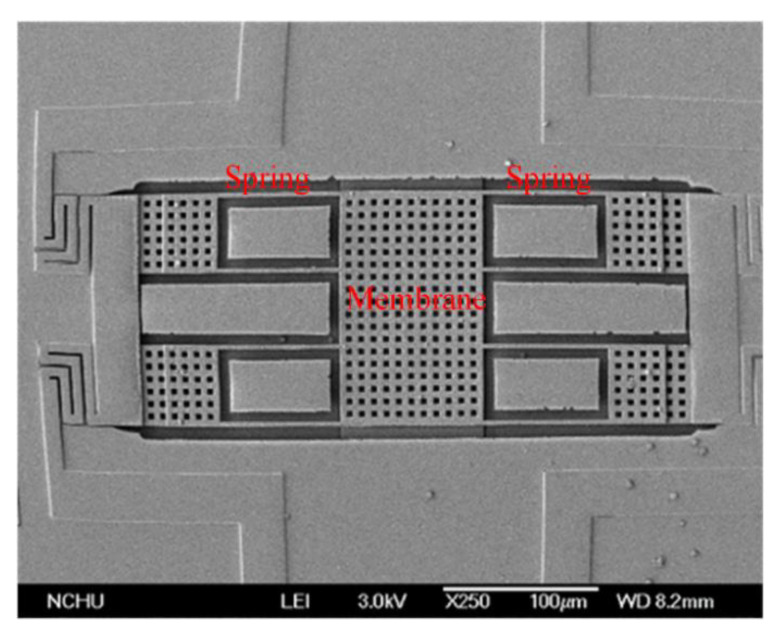
Scanning electron microscope (SEM) picture for the MSS.

**Figure 11:**
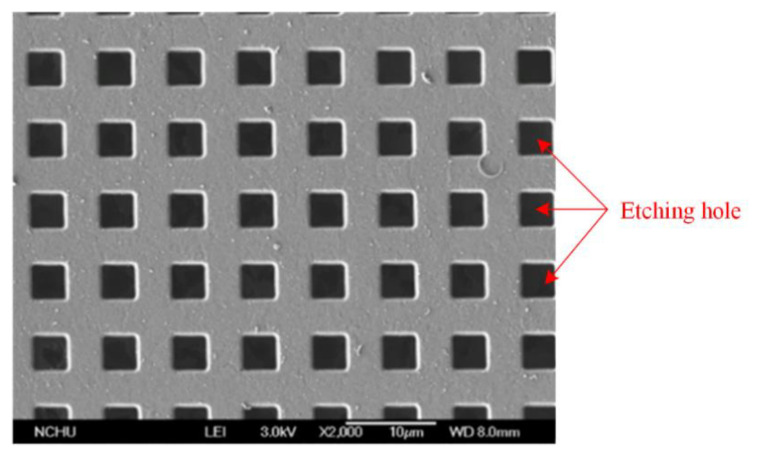
SEM picture of the membrane for the MSS.

Figure 8a illustrates the cross-sectional view for the MSS after the CMOS process. The membrane material was aluminum metal, and its thickness was about 1 μm. The spring material was the same as the membrane material, which was aluminum metal and about 1 μm thick. The anchors were a stack metal of tungsten and aluminum. The passivation layer was silicon nitride. An oxide layer that located under the membrane and springs must be etched, so that the membrane and springs became suspension structures. Then, a post-process was utilized to etch the oxide layer [29]. Figure 8b shows the MSS after completion of the post-process. A wet etching was used to remove the oxide layer. The etchant was buffer etch oxide (BOE) solution, which the etching rate for the oxide was 150 nm/min. The etching time for the post-process was about 27 min. As shown in Figure 8b, the suspension membrane and springs was released through the post-process. The structure image for the MSS was taken using a scanning electron microscope (JSM-6700F, Japan Electron Optics Laboratory Co. Ltd., Japan). Figure 10 shows the SEM picture for the MSS after completion of the post-process. The membrane and springs were released and became suspension structures. Figure 11 shows the SEM picture for the suspension membrane. The membrane had some etching holes, which decreased etching time in the post-process.

## 4. Results

An excellent radio frequency switch must have a high isolation in the off state and a low IL in the on state. The isolation and IL for the MSS were measured using an Agilent 8510C network analyzer and a Cascade probe station. The MSS was set in the Cascade probe station and connected with the Agilent 8510C network analyzer. The performance for the MSS in the on state was tested. The MSS was under without AV. The Agilent 8510C network analyzer measured the scattering parameter for the MSS. Figure 6 shows the measurement of IL for the MSS. The measured results showed that the MSS had an IL of 0.9 dB at 41 GHz. The measured and simulated results of the IL for the MSS are shown in Figure 6. The measured IL are in good agreement with the simulated IL.

The performance for the MSS in the off state was tested. The MSS was applied an AV of 10 V, so that the membrane moved down to the S line. The MSS was in the off state. The isolation for the MSS was recorded using the Agilent 8510C network analyzer. Figure 7 shows the measurement of isolation for the MSS. The measured results showed that the isolation for the MSS was 30 dB at 41 GHz. The measured and simulated isolation for the MSS are shown in Figure 7. The simulated isolation was 32 dB at 41 GHz. A comparison of both results, the measured isolation was lower 2 dB at resonance frequency than the simulated isolation.

Table 1 lists the insertion loss, isolation, and actuation voltage for various radio frequency microelectromechanical system switches. A MSS fabricated by Chae et al. [8] had an AV of 15.4 V, an isolation of 38.8 dB at 2.4 GHz and an IL of 0.23 dB at 2.4 GHz. Swarnkar et al. [9] presented a MSS, which the AV was 20 V. The isolation and IL for the MSS were 30 dB at 30 GHz and 0.5 dB at 30 GHz, respectively. A MSS proposed by Mafinejad et al. [10] had an AV of 18 V. The isolation for the MSS was 20 dB at 21 GHz and its IL was 1 dB at 21 dB. Anuroop et al. [11] fabricated a MSS using surface micromachining and a packaging technique. The IL for the MSS was 0.3 dB at 10.9 GHz, and the isolation for the MSS was 19.57 dB at 10.9 GHz. A MSS developed by Savin et al. [12] had an AV of 42 V. The IL for the MSS was 2 dB at 20 GHz and the isolation was 30 dB at 20 GHz. Rao et al. [14] employed surface micromachining to make a capacitive shunt MSS. The MSS had an AV of 5.5 V, an IL of 0.54 dB at 27 GHz and an isolation of 72.4 dB at 27 GHz. A MSS manufactured by Liu et al. [15] had an AV of 60 V. The isolation for the MSS was 36 dB at 40 GHz, and the IL for the switch was 0.43 dB at 40 GHz. Lin et al. [16] developed a MSS using the CMOS process. The MSS had an AV of 11 V. The IL and isolation for the MSS were 0.8 dB at 36 GHz and 19 dB at 36 GHz, respectively. A MSS presented by Yang et al. [17] was made using the CMOS process. The AV for the MSS was 11 V. The IL for the MSS was 0.85 dB at 35 GHz and the isolation was 25 dB at 35 GHz. In this work, the IL and isolation for the MSS were 0.9 dB at 41 GHz and 30 dB at 41 GHz, respectively. The AV for the MSS was 10 V. Comparing to Chae et al. [8], Swarnkar et al. [9], Mafinejad et al. [10], Savin et al. [12], Liu et al. [15], Lin et al. [16] and Yang et al. [17], the AV for the MSS in this work is lower than that of these MSS [8,9,10,12,15,16,17]. The isolation of this work exceeds that of Mafinejad et al. [10], Anuroop et al. [11], Lin et al. [16] and Yang et al. [17].

## 5. Conclusions

A radio frequency microelectromechanical system switch with a high isolation and a low pull-in voltage has been implemented utilizing the CMOS process and a post-process. The MSS had a sacrificial silicon dioxide layer after the CMOS process. In order to release the suspension structure in the MSS, the sacrificial silicon dioxide layer was removed by a wet etching post-process with BOE solution. The etching time for the post-process was 27 min. The post-process was without any mask and was compatible with the CMOS process, so that the fabrication for the MSS was simpler than that of these sensors [7,8,9,10,11,12,13,14,15] made by surface micromachining. The electrical properties for the MSS were simulated by the Agilent ADS tool. The simulation results depicted that the isolation for the MSS was 32 dB at 41 GHz and the IL for the MSS was 0.8 dB at 41 GHz. The experiments showed that the isolation for the MSS was 30 dB at 41 GHz and the IL for the MSS was 0.9 dB at 41 GHz. The measured isolation was lower 2 dB at resonance frequency than the simulated isolation. The CoventorWare software was used to evaluate the AV for the MSS. The simulation results revealed that the AV for the MSS was 10.5 V. The experiments showed the AV for the MSS was 10 V, which was in good agreement with the evaluated value. The actuation voltage for the radio frequency microelectromechanical system switch in this work was lower than that of Chae et al. [8], Swarnkar et al. [9], Mafinejad et al. [10], Savin et al. [12], Liu et al. [15], Lin et al. [16], and Yang et al. [17]. The isolation for the radio frequency microelectromechanical system switch in this work was higher than that of Mafinejad et al. [10], Anuroop et al. [11], Lin et al. [16], and Yang et al. [17].

## Figures and Tables

**Table 1:** Performances for various radio frequency microelectromechanical system switches.

Authors	Insertion Loss	Isolation	Actuation Voltage (V)
Chae et al. [8]	0.23 dB at 6 GHz	38.8 dB at 2.4 GHz	15.4
Swarnkar et al. [9]	0.5 dB at 30 GHz	30 dB at 30 GHz	20
Mafinejad et al. [10]	1 dB at 21 GHz	20 dB at 21 GHz	18
Anuroop et al. [11]	0.3 dB at 10.9 GHz	19.57 dB at 10.9 GHz	–
Savin et al. [12]	2 dB at 20 GHz	30.5 dB at20 GHz	42
Rao et al. [14]	0.54 dB at 27 GHz	72.4 dB at 27 GHz	5.5
Liu et al. [15]	0.43 dB at 40 GHz	36 dB at 40 GHz	60
Lin et al. [16]	0.8 dB at 36 GHz	19 dB at 36 GHz	11
Yang et al. [17]	0.85 dB at 35 GHz	25 dB at 35 GHz	11
This work	0.9 dB at 41 GHz	30 dB at 41 GHz	10.5

## Data Availability

Data sharing not applicable.
